# *Bacillus thuringiensis* and Chlorantraniliprole Trigger the Expression of Detoxification-Related Genes in the Larval Midgut of *Plutella xylostella*

**DOI:** 10.3389/fphys.2021.780255

**Published:** 2021-12-13

**Authors:** Muhammad Zeeshan Shabbir, Xiangbing Yang, Raufa Batool, Fei Yin, Paul E. Kendra, Zhen-Yu Li

**Affiliations:** ^1^Institute of Plant Protection, Guangdong Academy of Agricultural Sciences, Guangzhou, China; ^2^Guangdong Provincial Key Laboratory of High Technology for Plant Protection, Guangzhou, China; ^3^USDA-ARS, Subtropical Horticulture Research Station, Miami, FL, United States; ^4^State Key Laboratory for Biology of Plant Diseases and Insect Pests, Institute of Plant Protection, Chinese Academy of Agricultural Sciences, Beijing, China

**Keywords:** *Bacillus thuringiensis*, chlorantraniliprole, *Plutella xylostella*, resistance management, RNA sequencing, gene expression

## Abstract

**Background:** Diamondback moth (DBM), *Plutella xylostella* (L.), has developed resistance to many insecticides. The molecular mechanism of DBM resistance to Bt-G033A combined with chlorantraniliprole (CL) remains undefined.

**Methods:** In this study, field-resistant strains of *Plutella xylostella* to three pesticides, namely, *Bacillus thuringiensis* (Bt) toxin (Bt-G033A), CL, and a mixture of Bt + CL, were selected to evaluate the resistance level. Additionally, transcriptomic profiles of a susceptible (SS-DBM), field-resistant (FOH-DBM), Bt-resistant (Bt-DBM), CL-resistant (CL-DBM), and Bt + CL-resistant (BtC-DBM) strains were performed by comparative analysis to identify genes responsible for detoxification.

**Results:** The Bt-G033A was the most toxic chemical to all the DBM strains among the three insecticides. The comparative analysis identified 25,518 differentially expressed genes (DEGs) between pairs/combinations of strains. DEGs were enriched in pathways related to metabolic and catalytic activity and ABC transporter in resistant strains. In total, 17 metabolic resistance-related candidate genes were identified in resistance to Bt-G033A, CL, and Bt + CL by co-expression network analysis. Within candidate genes, the majority was upregulated in key genes including cytochrome P450, glutathione *S*-transferase (GST), carboxylesterase, and acetylcholinesterase in CL- and BtC-resistant strains. Furthermore, aminopeptidase N (APN), alkaline phosphatase (ALP), cadherin, trypsin, and ABC transporter genes were eminent as Bt-resistance-related genes. Expression patterns of key genes by the quantitative real-time PCR (qRT-PCR) proved the credibility of transcriptome data and suggest their association in the detoxification process.

**Conclusion:** To date, this study is the most comprehensive research presenting functional transcriptome analysis of DBM using Bt-G033A and CL combined insecticidal activity.

## Introduction

The diamondback moth (DBM), *Plutella xylostella* (L.) (*P. xylostella*) (Lepidoptera: Plutellidae), is a global pest of *Brassica* crops worldwide (Li et al., 2016; Jaleel et al., 2020). It can rapidly develop with extensive generation overlap and disperse quickly over substantial distances, which have made this pest particularly difficult to control. Currently, the control of *P. xylostella* is heavily dependent on pesticides causing several environmental problems including health issues and pollution and the development of resistance of the target insects (Pu et al., 2010; Yin et al., 2019). To the best of our knowledge, *P. xylostella* has developed resistance to over 95 insecticide compounds (Guo et al., 2013; Liu et al., 2015; Lima Neto et al., 2016). Although chlorantraniliprole (CL) is a novel diamide insecticide that is effective for control of *P. xylostella* (Guo et al., 2014), a field population of DBM has developed a 2,000-fold resistance to CL after 2 years of exposure in Southern China and Southeast Asia (Edralin et al., 2011; Wang and Wu, 2012). Also, the resistance level of DBM in field populations can vary among field sites, making resistance management more difficult (Wang et al., 2010). Therefore, effective management of this destructive pest is urgently needed.

Since repeated applications of conventional insecticides have resulted in a substantial increase in resistance in DBM populations, biological pesticides including Bt insecticides have been adopted as alternatives for managing Lepidoptera pests (Melatti et al., 2010; Shabbir et al., 2018; Prabu et al., 2020). However, DBM has rapidly developed significant resistance to various Bt toxins, although Bt insecticides showed negligible impact on non-target organisms (Jiang et al., 2015; Yin et al., 2016). Therefore, to cope with DBM resistance, rotation of insecticides with different modes of action has been utilized to lower the selection pressure (Zhao et al., 2010). To delay resistance evolution, combining various insecticides for target pests is necessary to ensure crop protection (Stemele, 2017; Yang et al., 2018; Zhao et al., 2018). Although different types of chemical combinations are commonly applied against lepidopteran pests (Cang et al., 2017; Zhao et al., 2018; Wang et al., 2019), studies on combinations of Bt products and insecticides have been little reported.

The evolution of resistance is a natural and unavoidable process. The mechanism of increased resistance to different insecticides is the enrichment of xenobiotic detoxification and target site mutation (James and Davey, 2009). The roles of major detoxification genes such as glutathione S-transferase (GST), carboxylesterases (CarE), and cytochrome P450 monooxygenases in metabolic resistance in lepidopteran pests have been widely reported (Li et al., 2007; Eziah et al., 2009; Pauchet et al., 2010). In addition, several Bt-binding proteins have been documented in *P. xylostella* (Gahan et al., 2010; Tiewsiri and Wang, 2011; Zhu et al., 2011). Previous studies have focused on mutations primarily correlated with insecticide resistance (Guo et al., 2014; Troczka et al., 2015). The role of mutation in diamide insecticide resistance has been confirmed by the CRISPR-Cas9-mediated genome editing in lepidopteran pests (Zuo et al., 2017). Similarly, genes (G4946E and I4790K) have also been associated with diamide resistance in DBM (Jouraku et al., 2020). Still, the occurrence of insecticide mutation is unclear, as information is insufficient about selecting ryanodine receptors (RyRs) allele in the field (Troczka et al., 2015). Thus, in-depth knowledge about the characterization of detoxification factors is essential for understanding the metabolic resistance mechanism to Cry toxins and insecticides in target pests.

Although the mechanism of resistance to insecticides in *P. xylostella* has been studied widely, complete information about the expression of genes linked to pesticides and Cry toxins is limited. To the best of our knowledge, no previous studies have addressed the molecular mechanism of resistance in *P. xylostella* by using a combination of Bt toxins and insecticides. A combination of Bt-G033A [*Bacillus thuringiensis* (Bt) subsp. *aizawai* G03, contains *cry1Ac*, *cry1Ac*, *cry1Ca*, and *cry2Ab* genes] and CL was used for the first time to determine the gene networks and molecular mechanism of resistance in *P. xylostella*. Recently, advancements in transcriptome analysis have provided several approaches for investigating the metabolic response of insects and comparing gene regulation in resistant and susceptible strains (Wang et al., 2010; Chen et al., 2011). In this study, we compared midgut tissues of susceptible (SS-DBM) and resistant strains [field-resistant (FOH-DBM), CL-resistant (CL-DBM), Bt-resistant (Bt-DBM), and Bt + CL-resistant (BtC-DBM)] of *P. xylostella* using transcriptome analysis to identify common genes that respond to different insecticides (CL, Bt-G033A, and mixture/combination of Bt and CL). Furthermore, the differentially expressed genes (DEGs) in DBM strains linked to metabolic resistance to Bt, CL, and Bt + CL (a mixture of Bt and CL) were identified and validated using qRT-PCR analysis. Current data will be useful for studying systemic differences between DBM strains and identifying genes that might confer resistance to Bt and CL.

## Materials and Methods

### Diamondback Moth Strains

A colony of *P. xylostella* was originally collected from the vegetable fields of Guangdong province of China in 2002 and reared without exposure to any insecticides under constant laboratory conditions of 25 ± 2°C, 70–80% relative humidity (RH), and a photoperiod of 16:8 h [light:dark (L:D)]. A high-resistant strain of DBM was collected from the Shijing county of Guangdong province in 2017 and named as FOH-DBM. Detailed information with respect to insecticide resistance of FOH-DBM is given in Supplementary Figure 1 and also discussed in our previous manuscript (Shabbir et al., 2021). Later, this population was established in the greenhouse and reared on cabbage without exposure to insecticide. The CL-DBM, Bt-DBM, and BtC-DBM strains were derived from FOH-DBM strain by two rounds of selection with CL, Bt (Bt-G033A), and mixture of Bt + CL, respectively (Shabbir et al., 2021). The rearing conditions in the greenhouse were maintained at 25 ± 2°C, 70–80% RH, and a photoperiod of 16:8 h (L:D). In September 2017, the greenhouse was divided into four compartments (1–4). Compartment 1 was used for maintaining DBM larvae with no treatment; compartment 2 was used to rear DBM on cabbage plants treated with Bt 4,000X, 1.5 g (Bt powder), and 6 L water; compartment 3 was used to rear DBM on cabbage plants sprayed with mixture treatment of Bt and CL 2,000–1,000X, 3 g (Bt powder), and 6 ml CL into 6 L water; and in compartment 4, DBM was reared on cabbage plants sprayed with CL 300X, 20 ml (5% CL) plus 6 L water. In March 2018, these four compartments were treated again as following: compartment 1, no treatment applied; compartment 2, sprayed cabbage plants with Bt 8,000X, 1.25 g (Bt powder), and 10 L water; compartment 3, sprayed plants with 16,000–3,200X, 0.625 g (Bt powder), 0.125 ml (5% CL), and 10 L water; and compartment 4, treated with CL 1,600X and 6.25 ml (5% CL) plus 10 L water (Shabbir et al., 2021).

### Insecticides

The chemicals used in this experiment included CL (200 g L^–1^ SC) purchased from the DuPont Agricultural Chemicals Ltd. (United States) and Bt-G033A, which was provided by the Huazhong Agricultural University, China.

### Leaf Bioassay

Leaf-dip bioassays were conducted to determine the resistance level of DBM from the CL, Bt-G033A, and Bt + CL treatment. Median lethal concentration (LC_50_) values were determined in DBM strains to compare the resistance level. For bioassay studies, we adopted the cabbage leaf-dip method of Tabashnik et al. (1987). Insecticides were dissolved in 100 ml distilled water and solutions of different concentrations were prepared with 0.1% Triton X-100. The second instar larvae from each strain were exposed to seven to eight concentrations of each insecticide. The concentrations ranging from 0.5 to 35 ppm for Bt, 10.5 to 700 ppm for CL, and 5.7 to 368 ppm for Bt + CL [17.5 ppm of Bt + 350 ppm of CL dissolved in 100 ml double-distilled water (ddH_2_O)] were used for bioassay. The concentrations (0, 0.03, 0.06, 0.013, 0.25, 0.50, 1, and 2 ppm) of Bt, CL, and Bt + CL were used for SS-DBM. Each dose was replicated three times for all the DBM strains. To conduct the leaf-dip bioassay, at each concentration, three cabbage leaf disks (*d* = 6 cm) were dipped in each insecticide solution for 15 s, and then air-dried for 2 h at room temperature. The control cabbage leaf disks were immersed in distilled water solution and then air-dried. The treated and control cabbage leaves were placed individually into petri dishes (2.5 cm H × 8.5 cm D). At each dose for each insecticide, 10 second instar larvae were placed on a treated leaf disk in plastic petri dish and kept at 25 ± 2°C and 65 ± 5% RH. Mortality was recorded after 48 h. The control mortality was also documented.

### Ribonucleic Acid Extraction, Library Construction, and Sequencing

The SS-DBM, Bt-DBM, CL-DBM, BtC-DBM, and FOH-DBM strains were selected to detect the resistance-related genes to Bt-G033A, CL, and Bt + CL, respectively. To induce resistance, these resistant strains were further treated with the highest doses of each insecticide by leaf-dipping test as discussed above. At 2 days posttreatment, treated DBM was collected for midgut extraction. A total of 50–60 larvae were collected for RNA extraction from midgut of each sample using the RNAprep Pure Kit DP432 (Tiangen Biotech, Beijing, China). Three biological replicates for each sample were collected and used for Illumina sequencing and gene expression analysis. RNA samples from all the DBM strains were evaluated for their stability using the Qsep1 instrument. 3 μg of total RNA was used to construct RNA libraries with the MGIEasy mRNA Library Prep Kit (Xu et al., 2019).

### Bioinformatics Analysis of RNA Sequencing

The adapter and low-quality reads were filtered through cutadapt (version 1.11). Clean reads were mapped to the contigs with paired-end reads by Hisat2 (version 2.1.0), allowing up to two mismatches (Su et al., 2019). These genes were subjected to alignment against public protein databases: Pfam (Pfam protein families) and UniProt (Swiss-Prot). It comprised RNA-seq by expectation-maximization (RSEM) (version 1.2.6) for transcript abundance estimation and normalization of expression values as fragments per kilobase of transcript per million mapped reads (FPKM) (Li and Dewey, 2011). DEGs were identified with DESeq2 with a filter threshold of adjusted *q*-value <0.05 and | log2 fold change| > 1 (Bakhtiarizadeh et al., 2019).

The clusterProfiler^1^ in R package (Yu et al., 2012) was employed to perform the Gene Ontology (GO) and the Kyoto Encyclopedia of Genes and Genomes (KEGG)^2^ enrichment analysis. The GO and the KEGG enrichment analysis were calculated using a hypergeometric distribution with a *Q* value cutoff of 0.05. *Q* value obtained by the Fisher’s exact test was adjusted with false discovery rate (FDR) for multiple comparisons (Liu et al., 2019).

### Co-expression Network Analysis of Genes

To explore the association of genes to insecticide resistance, co-expression network analyses were performed using a correlation calculator. Cytoscape software (3.7.2, Cytoscape, San Diego, CA, United States) was used to build the regulation network analysis for insecticide resistance-related genes. A threshold level of ≥2-fold, FDR ≥ 0.05 for upregulated and ≤−2-fold, and FDR ≥ 0.05 for downregulated genes were taken to construct the co-expression network map.

### Validation of Key Genes by Quantitative Real-Time PCR

Expression levels of key genes selected after co-expression network analysis were determined by the quantitative real-time PCR (qRT-PCR) in DBM strains. Total RNA was extracted from midgut of DBM larvae using the Easy-spin RNA Isolation Kit (Biomed, Beijing, China). Moloney murine leukemia virus (M-MLV) Reverse Transcriptase (Takara, Japan) was used for the first-strand complementary DNA (cDNA) synthesis. The primers were synthesized by the Invitrogen Trading (Shanghai) Corporation Ltd. The details of primers are shown in Supplementary Table 1. The qRT-PCR was performed according to our previous research protocol (Shabbir et al., 2020). Actin was used as a reference gene. The 2^−ΔΔ*CT*^ method was used to calculate the relative gene expression level in DBM strains.

### Statistical Analysis

The LC_50_ with fiducial limits and the chi-square (*x*^2^) values were determined by probit analysis using Probit Or LOgit (POLO-PC) LeOra software (Parma, MO, United States). The one-way ANOVA followed by the Tukey’s honestly significant difference (HSD) for multiple comparisons was used to analyze the significant expression of key genes in the qRT-PCR analyses. All the statistical analyses were carried out using the SPSS software (SPSS Inc., Chicago, IL, United States).

## Results

### Determination of Toxicity

The leaf bioassay indicated that the DBM populations showed variable degrees of resistance to insecticides tested (Bt-G033A, CL, and Bt + CL) (Table 1). The LC_50_ values for Bt-G033A, CL, and mixture insecticide (Bt + CL) were significantly higher in FOH-DBM and resistant strains (Bt-DBM, CL-DBM, and BtC-DBM) compared to SS-DBM strain. The resistance level of these DBM strains is different due to different generations of the strains from the results of our previous article (Shabbir et al., 2021). The results revealed that Bt-G033A was the most toxic to all the DBM strains among the tested insecticides. Concisely, the susceptibility level of DBM strains to tested chemicals from the most to least was Bt-G033A > Bt + CL > CL (Table 1).

**TABLE 1:** Median lethal concentration (LC_50_) of Bt-G033A, chlorantraniliprole, and Bt + chlorantraniliprole against five strains of *Plutella xylostella.*

**Insecticide**	**Strains**	**LC_50_ (95% FL) (mg/l)**	**df**	**χ^2^**	**RR^a^**
Chlorantraniliprole	SS-DBM^c^	0.189 (0.054–0.479)	5	1.505	–
	FOH-DBM^d^	7.485 (6.225–8.436)	5	0.744	39.6
	Bt-DBM^e^	93.691 (65.582–113.493)	5	1.642	495.7
	CL-DBM^f^	230.728 (151.731–383.323)	5	1.069	>1000
	BtC-DBM^g^	166.292 (109.621–342.673)	5	0.731	879.8
Bt-G033A	SS-DBM	0.053 (0.015–0.101)	5	1.230	–
	FOH-DBM	0.344 (0.003–0.361)	5	0.686	6.5
	Bt-DBM	2.294 (0.499–4.465)	5	0.627	43.3
	CL-DBM	0.871 (0.362–1.618)	5	4.577	16.4
	BtC-DBM	1.072 (0.351–1.979)	5	1.756	20.2
Bt + CL^b^	SS-DBM	0.141 (0.064–0.249)	5	2.043	–
	FOH-DBM	5.084 (3.696–7.506)	5	2.251	36.1
	Bt-DBM	30.289 (14.583–60.023)	5	3.395	214.8
	CL-DBM	24.398 (11.453–42.942)	5	1.403	173.1
	BtC-DBM	25.832 (18.919–35.853)	5	0.811	183.2

*^a^RR, resistance ratio = LC_50_ of a particular strain divided by LC_50_ of susceptible strain.*

*^b^Bt + CL is the insecticide mixture of Bt and chlorantraniliprole (1:1).*

*^c^SS-DBM is laboratory susceptible strain.*

*^d^FOH-DBM is the field high-resistant strain.*

*^e^Bt-DBM is the resistant strain treated with Bt-G033A.*

*^f^CL-DBM is the resistant strain treated with chlorantraniliprole insecticide.*

*^g^BtC-DBM is the resistant strain of DMB selected with a mixture of Bt and chlorantraniliprole.*

### Ribonucleic Acid Sequencing and Read Assembly

The cDNA samples of midgut tissue from resistant strains (FOH-DBM, Bt-DBM, CL-DBM, and BtC-DBM) and SS-DBM strain of *P. xylostella* were subjected to high-throughput Illumina sequencing to obtain an overview of gene expression profile. RNA sequencing from resistant and SS-DBM strains ranged from 118,757,080 to 160,256,478. The numbers of reads ranged from 60.61 to 64.71% and were mapped to trinity spliced transcriptomes. The GC contents ranged from 49.42 to 55.64% (Table 2).

**TABLE 2:** Summary of reads in resistant and susceptible strains of *Plutella xylostella* transcriptomes.

**Samples**	**Raw reads**	**Clean reads**	**Total mapped %**	**Clean ratio**	**Q30 (%)**	**GC (%)**
SS-DBM	160,430,796	160,256,478	62.41%	99.92%	93.29%	55.64%
FOH-DBM	150,250,928	149,997,438	54.94%	99.74%	93.68%	53.41%
Bt-DBM	138,498,530	138,101,022	64.71%	99.85%	92.87%	54.18%
CL-DBM	118,990,394	118,757,080	63.33%	99.80%	92.90%	51.73%
BtC-DBM	120,013,556	119,484,296	60.61%	99.70%	92.61%	49.42%

### Identification of Differentially Expressed Genes

**FIGURE 1:**
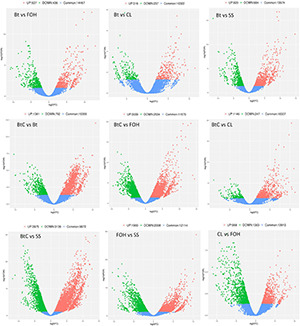
Differentially expressed genes (DEGs) (>2-fold change, FDR < 0.05) between pairwise comparisons of resistant and susceptible strains of *Plutella xylostella*. The scatter in the figure represents each gene. Red indicates upregulated genes, green indicates downregulated genes, and blue are common genes.

**FIGURE 2:**
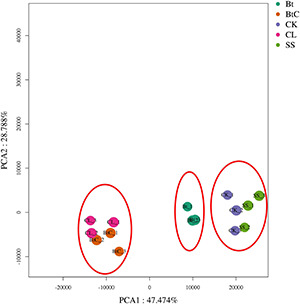
Principal component analysis (PCA) analysis of samples in transcriptomic analysis showing expression level of DEGs in all the DBM groups.

Pairwise comparisons were made to understand the expression patterns of genes between DBM strains. The DEGs (>2-fold change, FDR < 0.05) were identified in nine combinations of DBM strains. There were 973 DEGs (537 upregulated and 436 downregulated) in Bt-DBM vs. FOH-DBM groups; 553 (316 upregulated and 237 downregulated) in Bt-DBM vs. CL-DBM groups; 1,504 (820 upregulated and 684 downregulated) in Bt-DBM vs. SS-DBM groups; 2,173 (1,381 upregulated and 792 downregulated) in BtC-DBM vs. Bt-DBM groups; 5,573 (3,039 upregulated and 2,534 downregulated) in BtC-DBM vs. FOH-DBM groups; 1,392 (1,145 upregulated and 247 downregulated) in BtC-DBM vs. CL-DBM groups; 7,111 (3,975 upregulated and 3,136 downregulated) in BtC-DBM vs. SS-DBM groups; 3,908 (1,900 upregulated and 2,008 downregulated) in FOH-DBM vs. SS-DBM groups; and 2,331 (968 upregulated and 1,363 downregulated) in CL-DBM vs. FOH-DBM groups (Figure 1). These pair/group comparisons showed more upregulated DEG than downregulated genes, except for two pairs, BtC-DBM vs. SS-DBM groups and CL-DBM vs. FOH-DBM groups (Figure 1). Variability of data was checked by principal component analysis (PCA) analysis, which suggests that experimental data are reliable stable and can be used for further analysis (Figure 2).

### Gene Ontology and the Kyoto Encyclopedia of Genes and Genomes Pathway Enrichment

**FIGURE 3:**
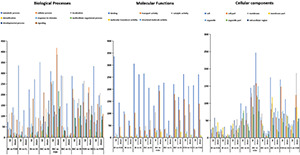
The Gene Ontology (GO) classifications of DEGs between pairwise comparisons following the treatment of Bt-G033A, chlorantraniliprole (CL), and Bt + CL insecticides. DEGs are classified into three categories: molecular function, cellular components, and biological process. The *Y*-axis represents the number of downregulation and upregulation of genes in each GO term.

The genes annotated in the GO classification were comprised of three major domains: biological process, cellular component, and molecular function. DEGs (*p* ≥ 0.05, FDR > 2) analysis revealed that 172, 24, 264, 78, 584, 4, 768, 287, and 258 DEGs were downregulated, whereas 75, 17, 61, 242, 70, 211, 91, 43, and 69 DEGs were upregulated in the pairs/combinations of DBM strains (Figure 3), respectively. Most genes affected by Bt-G033A and CL and their combination treatments were assigned to binding, transport activity, and catalytic activity in molecular function; cell, cell part, and signaling in the cellular process; and metabolic process, cellular process, and localization in biological process (Figure 3). DEGs analysis showed no noticeable difference when compared among different pairs of strains, except for BtC-DBM vs. FOH-DBM groups. This pair showed a relatively higher proportion of downregulated genes in terms of cellular process and localization than other pairs of strains (Figure 3).

**FIGURE 4:**
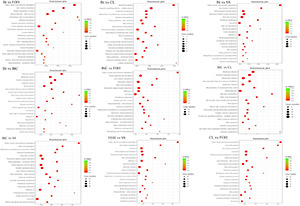
The Kyoto Encyclopedia of Genes and Genomes (KEGG) pathway enrichment scatter plot among pairwise comparison of Diamondback moth (DBM) strains. The horizontal axis represents the rich path plotted and horizontal axis represents path factor corresponding to rich factor. The size of *p*-value is represented by the color of the point. The numbers of genes in each pathway are expressed by the size of the point.

The KEGG analysis of DEGs identified from pairwise comparisons between susceptible and resistant strains provided information of pathways and gene functions associated with Bt and CL molecular mechanism (Supplementary Table 2). In the KEGG database, 20 highly enriched (*p* ≤ 0.05) pathways comprised of “valine, leucine, and isoleucine degradation,” “drug metabolism-cytochrome P450,” and “glutathione metabolism” in all the pairwise comparisons (Figure 4).

### Differentially Expressed Genes Following Insecticides Treatment

In the pairwise comparison between susceptible and treated larvae, we found detoxification genes such as cytochrome P450 monooxygenase, GST, acetylcholinesterase (AChE), CarE, glucuronosyltransferase, trypsin, nicotinamide adenine dinucleotide (NADH) dehydrogenase, and glutamate receptors potentially involved in insecticide resistance in pairs of DBM strains. Similarly, Bt resistance-related genes such as alkaline phosphatase, aminopeptidase N (APN), chitinase, cadherin, and ABC transporter were also identified (Supplementary Table 3).

The genes likely responsible for xenobiotic metabolism such as P450, CarE, AChE, and GST were more upregulated in CL-DBM vs. FOH-DBM groups with the rising of CL resistance level. Two upregulated putative insecticide target genes glutamate receptors were identified in the pair of CL-DBM vs. FOH-DBM comparison. The genes related to cuticle formation such as dehydrogenase and chitinase were mostly upregulated in pairs of DBM strains. Two downregulated RyR genes were identified in BtC-DBM vs. SS-DBM and CL-DBM vs. FOH-DBM pairs. Some immune-related genes such as serine protease were also found to be more upregulated in all the pairs of comparisons (Table 3).

**TABLE 3:** Differentially expressed genes that potentially involved in detoxification metabolism.

**Genes**	**Upregulated/Downregulated**								
	**Bt vs. FOH**	**Bt vs. CL**	**Bt vs. SS**	**Bt vs. BtC**	**BtC vs. FOH**	**BtC vs. CL**	**BtC vs. SS**	**FOH vs. SS**	**CL vs. FOH**
**Insecticide target genes**									
Cytochrome P450	4/3	5/3	10/6	3/8	11/8	6/4	16/8	14/10	22/9
Glutathione S-transferase (GST)	0/2	2/1	0/4	1/1	1/11	3/2	1/16	1/4	7/2
Carboxylesterase	0/1	1/2	2/1	1/3	2/7	2/1	3/7	0/1	6/2
bbAcetylcholinesterase (AChE)	1/0	2/1	0/2	6/5	2/12	2/1	2/13	1/10	10/4
NADH dehydrogenase	2/3	1/1	1/1	10/2	3/7	8/1	12/12	5/6	1/4
UDP-glucuronosyltransferase	3/2	3/0	1/3	1/6	1/8	1/2	2/8	0/2	5/3
Sodium channel	2/5	1/0	0/0	7/5	1/1	5/1	3/1	4/4	2/7
Glutamate receptors	0/1	0/2	1/3	1/0	6/1	0/1	5/1	3/0	2/0
neurotransmitter gamma-aminobutyric acid (GABA) receptors	0/0	1/0	1/3	1/2	1/5	0/0	0/5	0/4	0/1‘
Ryanodine receptor	0/0	0/0	0/0	0/0	0/0	0/0	0/1	0/0	0/1
Trehalose transporter	0/9	1/2	7/5	3/1	2/6	9/2	12/10	5/15	1/8
Catalase	0/0	2/1	4/0	1/2	1/3	4/0	4/2	1/3	0/5
N-acetylgalactosaminyltransferase	0/0	4/2	0/0	6/1	5/2	6/1	9/2	3/2	0/2
ATPase synthase subunit	1/1	1/1	3/7	12/3	4/3	2/2	8/7	8/10	3/2
Serpin	2/0	3/2	2/0	1/0	4/1	1/1	6/1	9/0	4/0
Serine protease	7/3	5/0	4/1	1/0	5/0	6/0	4/2	2/0	6/8
**Bt target genes**									
Cadherin	2/1	1/0	3/1	2/1	2/2	2/1	2/3	0/2	0/0
Aminopeptidase N	0/6	1/3	2/6	1/10	3/8	1/2	3/7	3/10	0/8
Trypsin	16/8	10/6	6/11	14/11	17/5	9/8	9/7	6/10	3/14
Alkaline phosphatase	2/0	1/0	1/0	1/0	2/1	0/0	0/2	0/1	0/0
ABC transporter	0/2	1/1	0/5	1/3	1/14	4/1	5/12	1/10	1/11
Chitinase	5/3	1/1	7/2	1/1	6/1	2/0	5/2	3/2	4/2

*Genes were identified in pairwise comparison of *Plutella xylostella* strains.*

*Differentially expressed genes are upregulated (*FDR* ≥ 0.05, logFC > 2-fold) and downregulated (*FDR* ≥ 0.05, FDR > −2-fold) between susceptible and resistant strains of Plutella xylostella.*

Bt resistance-related genes, especially aminopeptidase N and ABC transporter, were downregulated. In the pair of Bt-DBM vs. FOH-DBM, trypsin genes were mostly upregulated, whereas more downregulated genes were identified in the pair of Bt-DBM vs. SS-DBM comparison. Similarly, we identified two alkaline phosphatase (ALP) genes that were upregulated in the pair of Bt-DBM vs. FOH-DBM and one upregulated in the pair of Bt-DBM vs. SS-DBM. However, two of these genes were upregulated and one was downregulated in the pair of BtC-DBM vs. FOH-DBM. Chitinase and cadherin genes were more upregulated in the pairwise comparisons of DBM strains (Table 3).

### Co-expression Network Analysis of Genes Related to Resistance

**FIGURE 5:**
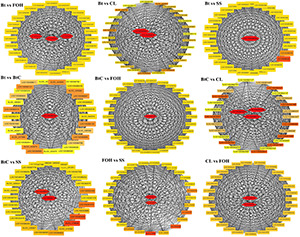
Gene networks and key genes involved in metabolic resistance among pairwise comparison of DBM strains identified by weighted correlation network analysis (WGCNA). Key genes within (highlighted with red) each network are related to insecticide target and metabolism.

To identify the key genes associated with resistance to Bt-G033A, CL, and Bt + CL, DEGs from all the pairs were subjected to co-expression network analysis. Based on the DEGs, a total of 30 highly enriched metabolic detoxification enzymes potentially involved in insecticide resistance were identified from all the pairs after the insecticide treatments. Furthermore, annotations of all these selected DEGs were obtained from the RNA-seq database to screen candidate genes that contributed to major resistance increase in each pairwise comparison (Figure 5).

The pairwise comparison of DBM strains revealed that most of the hub genes involved in metabolic detoxification were upregulated. We identified key genes such as CarE in pair of Bt-DBM vs. BtC-DBM, AChE in pair of BtC-DBM vs. CL-DBM, GST in pairs of BtC-DBM vs. SS-DBM and FOH-DBM vs. SS-DBM, whereas cytochrome P450 was identified in pairs of CL-DBM vs. FOH-DBM, BtC-DBM vs. CL-DBM, and BtC-DBM vs. SS-DBM. We identified trypsin upregulated in pairs of Bt-DBM vs. CL-DBM and BtC-DBM vs. CL-DBM, but downregulated in CL-DBM vs. FOH-DBM pair. Similarly, multidrug resistance genes, ABCC4, were downregulated key genes identified in the pairs of BtC-DBM vs. SS-DBM and FOH-DBM vs. SS-DBM, respectively (Supplementary Table 4).

### Confirmation of Key Genes by Quantitative Real-Time PCR

**FIGURE 6:**
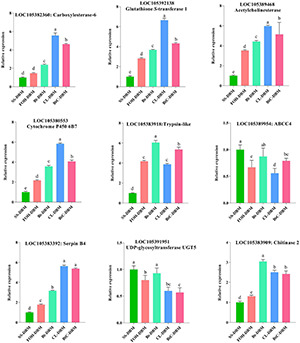
The quantitative real-time PCR (qRT-PCR)-based validation of selected key genes from susceptible and resistant strains of *Plutella xylostella*. Same letter on top of the bars indicated no significant difference was found [general linear model (GLM), least significant difference (LSD), *p* < 0.05].

Nine key genes, related to insecticide resistance and identified in pairs of DBM strains, were subjected to qRT-PCR analysis to validate the findings from RNA-seq data. The expression patterns of all the tested genes confirmed the trend with the data obtained from transcriptome analysis (Figure 6). Among the key genes, CarE, GST, cytochrome P450, AChE, and serpin were highly expressed in CL-induced DBM strain compared to other strains. Chitinase and trypsin showed higher expression levels in larvae treated with Bt-G033A. The expression level of genes encoding for ABCC4 and UDP-glycosyltransferase UGT5 in Bt-induced DBM was not significantly different compared to susceptible strain. In general, our results revealed that the expressions of detoxifying genes were higher in resistant strains than those insusceptible- and FOH-resistant strains (Figure 6).

## Discussion

The rapid evolution of resistance to pesticides and Cry toxins is a major challenge for managing lepidopteran pests including *P. xylostella* (Tabashnik et al., 2013). Assessment of insect resistance becomes necessary to guide appropriate implementation of chemical control and Bt products to understand the molecular mechanisms underlying development of resistance. In this study, we collected a DBM strain from one location and subjected it to two rounds of selection with Bt-G033A, CL, and a combination of the two insecticides. The resistant level of DBM strains to three tested insecticides from the most to the least ranked as Bt-G033A > Bt + CL > CL. Our results indicated that the combination of insecticides did not increase efficacy against DBM strains compared to Bt-G033A applied alone. These results are consistent with previous reports that a combination of two toxins may not delay resistance increase in lepidopteran pests as much as a single toxin in resistance management (Yang et al., 2018; Wang et al., 2019). However, the development of resistance under selection of a mixture of insecticides is dependent on multiple factors including the mode of inheritance of resistance to each insecticide (Tabashnik, 1989).

The molecular mechanism underlying the resistance to Bt-G033A, CL, or their mixture has not been fully understood yet. In this study, we investigated the molecular resistance mechanism for Bt-G033A, CL, and their combination in susceptible and resistance strains of DBM through transcriptomic analyses. We obtained a total of 25,518 DEGs from the pairs/comparisons of susceptible and resistant DBM strains. Comparative transcriptomic analysis showed more upregulation of DEGs in pairwise comparisons with the exception of two pairs (BtC-DBM vs. SS-DBM and CL-DBM vs. FOH-DBM) (Figure 1). Similarly, a previous study also reported more upregulated genes in Cry1Ac-resistant strain of DBM were identified (Lei et al., 2014).

The overall GO analysis identified a high proportion of genes related to cellular process, metabolic process, binding and localization, response to stimulus, transport activity, and catalytic activity. Mostly DEGs related to these categories had upregulated expression in pairwise comparisons (Figure 3). As these GO terms may be linked to detoxification xenobiotic process, the upregulation of these DEGs in treated larvae is responsible for resistance evolution. The KEGG analysis between pairwise comparisons of DBM strains showed that genes expression level was enriched in metabolic pathways. The genes involved in pathways such as glutathione metabolism and drug metabolism cytochrome P450 were upregulated (Figure 4). Other genes involved in signaling pathways and muscle control pathways were also identified as upregulated in pairs of BtC-DBM vs. Bt-DBM and BtC-DBM vs. CL-DBM. These results are consistent with a previous study, which revealed in CL-resistant *P. xylostella* strain, that upregulation of genes was involved in metabolic pathways such as drug metabolism and xenobiotic enzyme metabolism (Lin et al., 2013). Similarly, in the Cry1Ac-resistant *P. xylostella* strains, most DEGs involved in metabolic pathways and drug metabolism were upregulated (Lei et al., 2014). These results suggest that multiple genes involved in metabolic pathways, muscle contraction pathways, and drug metabolism pathways play dominant roles in resistance to Bt-G033A and CL.

In this study, detoxification genes were identified from insecticide-treated larvae. Detoxification of insecticides occurs in all the insects and involved several enzymes encoded for GST, P450, and Carboxylesterases (COE) families (Li et al., 2007). The genes related to insecticide resistance such as GST, cytochrome P450, and CarE were identified by the pairwise comparison of DBM strains (Supplementary Table 3). Cytochrome P450s are important detoxification enzymes, which are involved in xenobiotic metabolism and act on substrates to reduce the toxicity (Zimmer et al., 2014). In *P. xylostella*, cytochrome P450 was linked with resistance to abamectin and tebufenozide (Qian et al., 2008). In this study, most DEGs of cytochrome genes (31) were identified in the pair of CL-DBM vs. FOH-DBM strain (Table 3). Other more abundant genes of detoxification were identified in pair of CL-DBM vs. FOH-DBM, including GST (9), CarE (8), and AChE (14), and they were mostly upregulated. The identified genes encoding P450, GST, AChE, and CarE were less abundant in the CL treatment than those in the Bt-G033A or mixed insecticide treatments (Table 3). Previously, a study reported that cytochrome family genes (*Cyp301a1 and Cyp9e2*) were overtranscribed responding to insecticide treatment in a *P. xylostella* strain (Gao et al., 2018). Thus, increased expression of these detoxification genes might link directly to CL resistance in *P. xylostella* strains. Overexpression of the *CYP6BGI* gene was reported in a permethrin-resistant *P. xylostella* strain (Bautista et al., 2009). Likewise, overexpression of *P450s* genes was associated with neonicotinoid resistance in *Bemisia tabaci* (Karunker et al., 2008) and led to deltamethrin resistance in *Tribolium castaneum* (Zhu et al., 2010). Furthermore, the detoxification enzyme GST was reported in *P. xylostella* to confer resistance to CL, organophosphates, and chlorfluazuron insecticides (Sonoda and Tsumuki, 2005; Lin et al., 2013). CarE and GST were also reported to be involved in fufenozide resistance (Tang et al., 2011). The expression of detoxification genes to all the pairs of different insecticide treatments in this study suggests that these genes are likely responsive to insecticide resistance and xenobiotic detoxification process.

Several genes such as serpin, serine protease, GABA receptors, and glutamate receptors that may contribute to insecticide resistance were also upregulated in most treated pairs of DBM strains, but not all. These results are consistent with a previous study on identification of insecticide target genes including GABA receptor, AChE, nicotinic acetylcholine receptors (nAChRs), and RyR in the asian corn borer (ACB) transcriptome (Cui et al., 2017). AChEs are the principal targets of carbamates and organophosphate insecticides in insects, reflecting their role in neurotransmission. GABA receptors are a members of the cys-loop neurotransmitter receptors and the GABA-regulated chloride channel plays an important role in phenylpyrazole and organochlorine insecticides (Hosie et al., 1995). Some genes involved in cellular catabolism such as serine protease and trypsin were also identified in this study. The downregulated gene related to cuticular in CL-treated DBM indicated that this insecticide might play a role in thinning the cuticles in DBM. These thinned cuticles will help to accelerate the transportation of CL insecticide to the target site and improve its efficacy against the pest.

In this study, we identified two RyR genes with downregulation patterns: one was identified from the BtC-DBM vs. SS-DBM pair and the other from the CL-DBM vs. FOH-DBM comparison. A previous study reported that RyRs are major factors for *P. xylostella* resistance to CL (Troczka et al., 2012). As RyRs control the muscle contraction/excitation, it is speculated that the downregulation of RyRs is likely to reduce muscle excitability. Since the RyR was also identified in DBM treated with the mixture of Bt-G033A and CL, it remains to be determined whether the receptor is downregulated by the mixture. Also, NADH dehydrogenase genes were found more downregulated in the treatment of CL.

Bt resistance-related genes encoding for APN, ALP, cadherin, chitinase, trypsin, and ABC transporter were identified in this study (Supplementary Table 2). Most of *APN* genes were downregulated in Bt-resistant strains, whereas cadherin was upregulated in most pairs when compared with susceptible and FOH-DBM strains (Table 3). Downregulation of *APN* genes is involved in Bt resistance to different Cry toxins (Zhang et al., 2017; Shabbir et al., 2019). Different isoforms of APN, ALP, and cadherin are involved with different Cry toxin resistance (Flannagan et al., 2005; Pigott and Ellar, 2007). ABC transporter genes associated with drug resistance were also identified and most of them were downregulated in all the pairs of treated DBM compared to susceptible strains. ABC transporters identified in both the susceptible and resistant DBM strains included ABCC4, ABCC10, ABCB1, and ABCC1 in this study. Previous studies reported that ABC transporter (ABCB1) had been linked to multidrug resistance in mammalian systems (Shanker et al., 2010) and ABCC2 was linked with Cry1Ac resistance in lepidopteran insects (Gahan et al., 2010; Baxter et al., 2011). However, further studies are needed to determine the downregulation of these genes in DBM treated with Bt-G033A and the mixture insecticide.

We identified 17 gene networks, highly correlated with metabolic detoxification, muscle contraction pathways, and drug metabolism (Supplementary Table 4 and Figure 5). We identified key genes including cytochrome P450, CarE, GSTs, and UDP-glycosyltransferase UGT5 related to insecticide metabolism from the network built from top DEGs. ABC transporter was previously found to be related to Bt resistance (Gahan et al., 2010). Trypsin is considered as the main proteinase involved in Bt toxin activation and detoxification (Liu et al., 2015). We selected nine detoxification-related key genes to analyze their gene expression in DBM strains. The results showed that CarE, GST, AChE, cytochrome P450, and serpin genes were expressed significantly higher in response to CL followed by the mixture insecticide (Figure 6). These results further validated the association of these genes with CL detoxification. Chitinase and trypsin were highly expressed in the Bt-G033A treatment. Furthermore, our findings suggested that multiple genes involved in drug metabolism, muscle control, and metabolic pathways that play dominant roles in CL and Bt-G033A detoxification. Further investigations are needed to explain the phenomenon of upregulated or downregulated genes in CL and Bt-treated DBM, so as to provide practical guidance for Bt-G033A and CL resistance management of DBM.

*Bacillus thuringiensis* subsp. *aizawai* and the binary combination of Bt-G033A with CL can delay resistance development in *P. xylostella* compared to CL alone. To date, this study is the most comprehensive study presenting functional transcriptome analysis of DBM using the combined insecticidal activity of Bt-G033A and CL. Comparative transcriptome analysis of three insecticides enabled the identification of commonly responding genes involved in drug metabolism and the xenobiotic detoxification process. The prominent genes identified in this study include cytochrome P450, GST, and CarE with more upregulation and downregulation of RyRs in the CL treatment. Bt and the mixture insecticide are involved in DBM metabolic and catalytic pathways with the downregulation and upregulation of cadherin, APN, ALP, and ABC transporter genes. Considering the physiological functions of key/hub genes identified through co-expression network analysis, their downregulation or upregulation appears to be involved in the direct or indirect detoxification process. The expression of these key genes further supported our findings. The common response of key DEGs to three tested insecticides via pairs/combinations of DBM strains suggests their roles of key candidates in catalytic, metabolic, and drug xenobiotic detoxification pathways. Consequently, precise identification of such key genes following exposure to Bt and CL insecticides could serve as a landmark for searching metabolic factors to provide information on improving resistance management of DBM.

## Data Availability Statement

The datasets presented in this study can be found in online repositories. The names of the repository and accession number(s) can be found below: BioProject (https://www.ncbi.nlm.nih.gov/bioproject), PRJNA785284.

## Author Contributions

MZS and Z-YL conceived and designed the research. MZS conducted the experiment and drafted the original manuscript. MZS, RB, and FY analyzed the data. Z-YL, XY, and PK reviewed and edited the manuscript. All authors read and approved the final manuscript.

## Conflict of Interest

The authors declare that the research was conducted in the absence of any commercial or financial relationships that could be construed as a potential conflict of interest.

## Publisher’s Note

All claims expressed in this article are solely those of the authors and do not necessarily represent those of their affiliated organizations, or those of the publisher, the editors and the reviewers. Any product that may be evaluated in this article, or claim that may be made by its manufacturer, is not guaranteed or endorsed by the publisher.

## Footnotes

1
http://bioconductor.org/packages/release/bioc/html/clusterProfiler.html

2
http://www.genome.jp/kegg/
