# Supplementary figures and images for: Bacillus thuringiensis and Chlorantraniliprole Trigger the Expression of Detoxification-Related Genes in the Larval Midgut of Plutella xylostella
Source: Front Physiol. 2021 Dec 13;12:780255. doi: 10.3389/fphys.2021.780255 (PMC8710669; doi:10.3389/fphys.2021.780255)

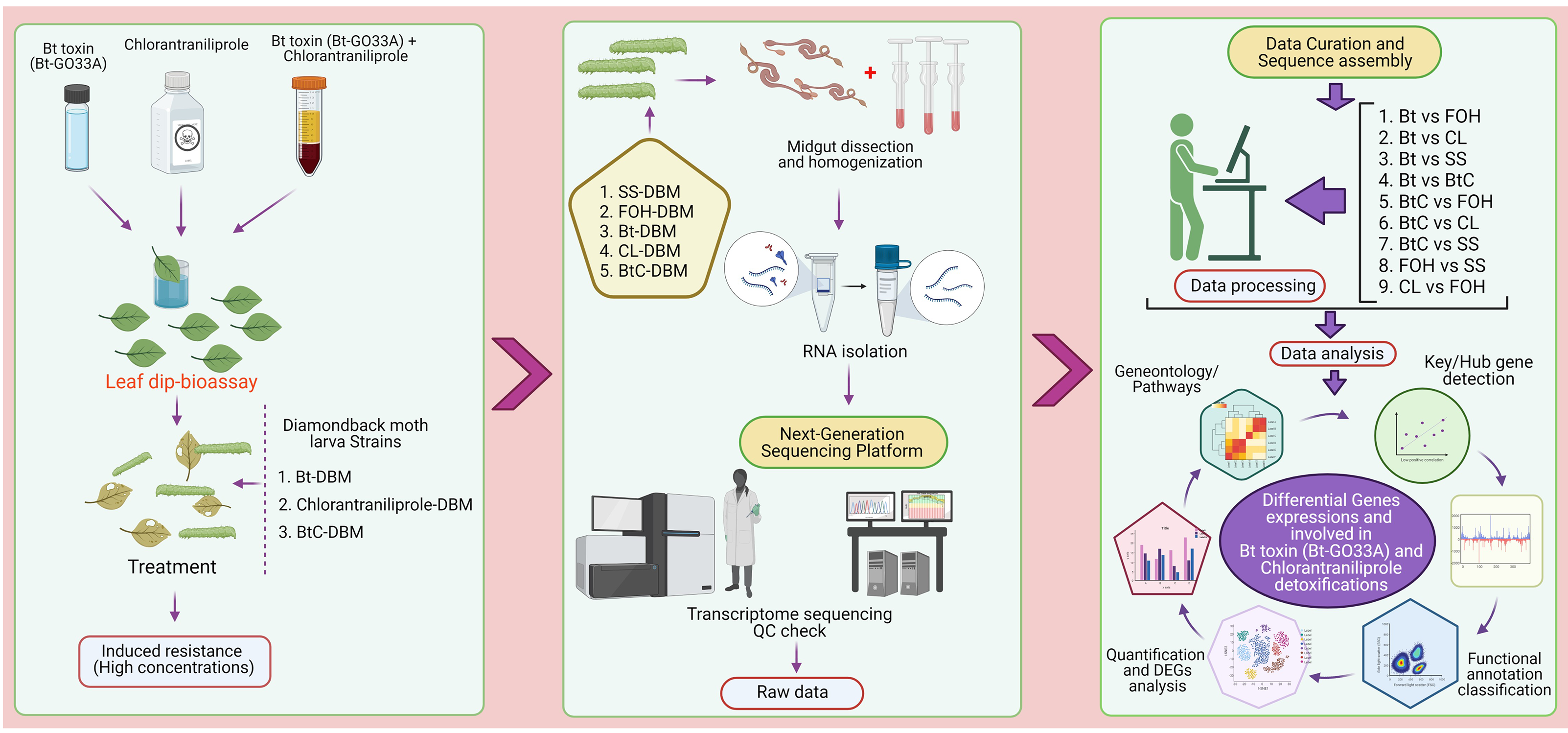

Supplement: Supplementary file 5 [file Image_1.TIF]
